# Tryptophan Metabolism ‘Hub’ Gene Expression Associates with Increased Inflammation and Severe Disease Outcomes in COVID-19 Infection and Inflammatory Bowel Disease

**DOI:** 10.3390/ijms232314776

**Published:** 2022-11-26

**Authors:** Sonia Bustamante, Yunki Yau, Victoria Boys, Jeff Chang, Sudarshan Paramsothy, Aviv Pudipeddi, Rupert W. Leong, Valerie C. Wasinger

**Affiliations:** 1 Bioanalytical Mass Spectrometry Facility, Mark Wainwright Analytical Centre, The University of New South Wales, Sydney, NSW 2052, Australia; 2 Department of Gastroenterology, Concord Repatriation General Hospital, Sydney, NSW 2139, Australia; 3 School of Medical Sciences, Faculty of Medicine, The University of New South Wales, Sydney, NSW 2052, Australia

**Keywords:** COVID-19, SARS-2, mitochondrial dysfunction, leaky-gut, NAD, PARP, WARS, IFN, tryptophan, IBD

## Abstract

The epithelial barrier’s primary role is to protect against entry of foreign and pathogenic elements. Both COVID-19 and Inflammatory Bowel Disease (IBD) show commonalities in symptoms and treatment with sensitization of the epithelial barrier inviting an immune response. In this study we use a multi-omics strategy to identify a common signature of immune disease that may be able to predict for more severe patient outcomes. Global proteomic approaches were applied to transcriptome and proteome. Further semi- and relative- quantitative targeted mass spectrometry methods were developed to substantiate the proteomic and metabolomics changes in nasal swabs from healthy, COVID-19 (24 h and 3 weeks post infection); serums from Crohn’s disease patients (scored for epithelial leak), terminal ileum tissue biopsies (patient matched inflamed and non-inflamed regions, and controls). We found that the tryptophan/kynurenine metabolism pathway is a ‘hub’ regulator of canonical and non-canonical transcription, macrophage release of cytokines and significant changes in the immune and metabolic status with increasing severity and disease course. Significantly modified pathways include stress response regulator EIF2 signaling (*p* = 1 × 10^−3)^; energy metabolism, KYNU (*p* = 4 × 10^−4^), WARS (*p* = 1 × 10^−7^); inflammation, and IDO activity (*p* = 1 × 10^−6^). Heightened levels of PARP1, WARS and KYNU are predictive at the acute stage of infection for resilience, while in contrast, levels remained high and are predictive of persistent and more severe outcomes in COVID disease. Generation of a targeted marker profile showed these changes in immune disease underlay resolution of epithelial barrier function and have the potential to define disease trajectory and more severe patient outcomes.

## 1. Introduction

Cytokine release is a two-edged sword that in some patients can lead to recovery, while others are left devastated by systemic inflammatory response and organ failure. This difference in immune control has played out on a global scale with the Coronavirus Disease (COVID) pandemic, with more severe clinical outcomes associated with patients suffering other health co-morbidities or advanced age [1]. However, recent evidence suggests that immune control is far more complex and dynamic [2] and may be better defined by immune cell status [3] and cellular exhaustion [4] to better explain therapeutic failure in some cases.

We reasoned that the discrepancy between disease resolution and persistence is related to trafficking, sabotage, and theft of the global cellular currency-energy. Both COVID-19 and IBD share symptoms and treatment regimes, indicating the underlying pathology and biochemical processes are common. Cytokine release is prevalent in both diseases and involves a combination of innate and adaptive immune control by recognition of harmful molecules and registering an appropriate response. Therefore, identification of biomarkers predictive of a more severe disease outcome may allow real-time tailored treatment decisions to be made for ‘at risk’ patients in both these acute and long/chronic conditions.

Evidence has shown that the asymptomatic recovery amongst COVID-19 sufferers can vary greatly with 60% of a 2904 Australian cohort recovered 3 weeks post infection (3 wpi) [5], while the more debilitating long-COVID (>2 months) and multisystem inflammatory syndrome (~2–6 wpi) are additional complications of SARS with similarities to other post-infectious syndromes and immune activation resulting in dysregulation of immune control [6]. Many of the clinical manifestations of COVID-19 infection and IBD are similar. This includes symptoms of lethargy and gastrointestinal involvement. Over 38% of severe COVID infections [7] and 29–37% of IBD sufferers experience diarrhea and IBS-like symptoms during the course of the disease [8]; with a higher prevalence in Crohn’s disease (CD) than Ulcerative colitis, with increased duration of symptoms associated with a worse prognosis in COVID infection [9]. IBD draws on many of the features triggered by pathogen infection seen in COVID-19, with similarities in host pattern recognition receptor (PRR) identification of pathogens or damage associated molecular patterns (DAMPs) and release of TNF-α, IFN-γ, IL-6, IL-17 and NfκB by macrophage TLR4 receptor pathways, and a concomitant reduction of T-regulatory cells (Treg). This allows for common immune suppressive and biological treatment options with utility across IBD and COVID [10]. In both diseases, fibrosis is potentially a severe outcome of damaged barrier tissues and inflammation that can lead to complications. For both COVID and IBD, treatment will involve the management of inflammation and restoration of epithelial barrier function at the sub clinical level to achieve resolution of infection and/or remission.

In this study, we utilize a multi-omics strategy to better understand the pathophysiology of immune triggered diseases. Our objective was to determine significant changes in both proteomic and transcriptomic profiles which were then used to determine the common hallmark genes and pathways of immune disruption. Secondly, a targeted assessment of key protein and metabolite regulators associating with changes in these pathways were applied to highlight more severe outcomes for both chronic and acute infections. Testing was performed across different sample types including biopsied tissue from the terminal ileum (TI), serums, and nasal swabs to demonstrate an applicability with a far broader reach than IBD and COVID.

## 2. Results

Often, mRNA is used as the proxy for protein abundance. During inflammation, timing and protein activation is an important and often overlooked aspect that can contribute to paradoxical fluctuations between transcripts, active proteins and their metabolic products. We therefore assessed the transcriptome, proteome and metabolome of patients with chronic and acute disease.

### 2.1. The Stress Response and Disturbances in Central Energy Metabolism Are Hallmarks of COVID-19 and IBD

**Figure 1:**
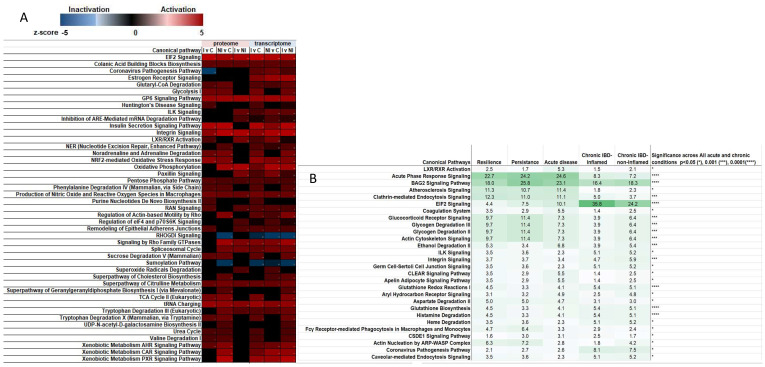
Enriched functional pathways in IBD and in common with SARS2 infection. (**A**) Proteome and transcriptome comparison of fold change between Inflamed and control, non-inflamed and control and inflamed and non-inflamed TI biopsy in chronic autoimmune CD patients. Pathways with significance in modulation with *p* < 0.001 (−log*p* > 3 in at least 1 group) and z-activation scores >2 or inactivation scores of <−2 (in at least 1 group) are shown. (**B**) Significantly modulated canonical pathways shared across all groups of acute disease (COVID positive acute, persistent and resolving groupings), and chronic disease (IBD inflamed and non-inflamed biopsy tissue). Green intensity represents higher significance within each grouping, while (*) represents significance across all groupings.

**Figure 2:**
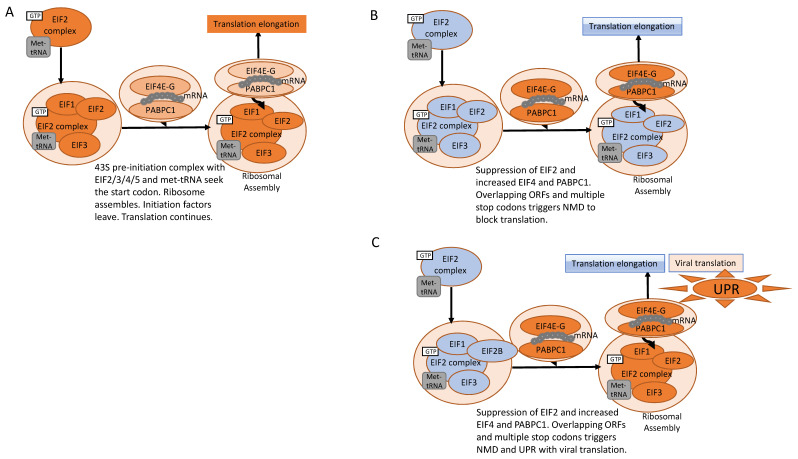
Longitudinal EIF signaling modulations in acute and chronic immune stimulated illness. (**A**) EIF2A activation in inflamed tissue (IBD), with limited EIF4A,G activation, (**B**) Repression of host translation but heightened expression of EIF4A,G in acute phase infection COVID-19, (**C**) translation initiation promoted in Persistent cases at 3 wpi (Resilient cases at 3 wpi have a dampened EIF2, EIF4 signaling stress response). Orange is indicative of activation, blue indicates inhibition. Intensity of colour represents level of activation or inhibition.

**Figure 3:**
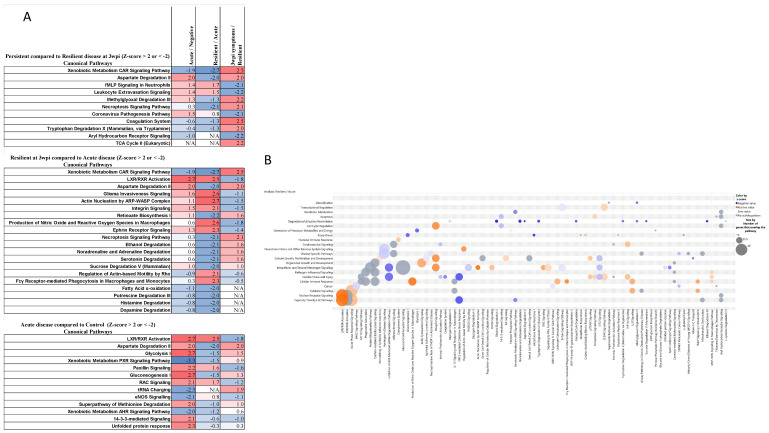
Proteome comparison of fold change between Acute disease (within 24 h COVID positive affirmation), Resilient (no symptoms 3 weeks post COVID infection), Persistent (3 weeks post infection-secondary infection or symptoms remaining). (**A**) Pathways with significance in activation z-activation scores ≥2 or inhibition scores of ≤−2 (in at least 1 group) are shown. (**B**) Canonical Pathway scores plotted as pathway names versus category [13]. The predicted effects of resilience and recovery from COVID at 3 wpi time-point clusters significantly on Canonical Pathway activity in Nasal epithelium. The bubbles are coloured according to predicted activation (orange = activation, blue = inhibition) z-score, and the sizes of the bubbles increase with significance based on the -log(*p*-value), Benjamini Hochberg-corrected right-tailed Fisher’s exact test scores. These data show that Xenobiotic insult, Nuclear Receptor and Cytokine signalling are the causal associations affecting pathways and indicate the association to clinical and pathological endpoints.

Global analysis revealed the stress response can be triggered by unfolded proteins, general amino acid starvation, and infections. Amino acid deprivation leads to an inbalance in tRNA charging (Figure 1) and has a direct effect on the regulation of translation initiation via the EIF2 signalling pathway. Translation of celluar and viral mRNA is controlled by EIF2. In addition to proteotoxic stress, Type 1 and 3 interferons also activate the EIF2 cascade to activate T-cells and trigger a tryptophan shortage, while translocation of various nuclear transcription factors assume control of transcription processes [11]. In fold change comparisons amongst inflamed and non-inflamed TI tissue, EIF2 signalling was universally activated (Figure 1A) and persists regardless of IBD inflammatory status. In further proteomic comparisons between IBD and acute SARS infected NE swab samples, the EIF2 signalling cascade was amongst the most significantly modulated pathways across all comparisons (Figure 1B, *p* < 0.0001), but in particular for acute SARS infection, while suppression of EIF2 signaling occurred in acute infection with increased levels of EIF4 and PABPC1 observed, EIF2 signalling remained higher in patients with ongoing symptom (Figure 2C). Sensing of long untranslated regions with overlapping ORF’s and multiple stop codons as is typified in SARS dsRNA genome, triggers non-sense mediated decay (NMD) to block viral replication [12]. However, NMD suppression induces the UPR (Figure 2 and Figure 3) allowing viral sabotage, by host cleavage of the PABP1 and EIF4G, impeding host translation while viral NSP1 blocks binding to the 40S ribosome thereby enhancing viral translation.

Infection elicits an immune response via PAMPs and DAMPs which are secreted by immune cells or passively released by injured or dying cells. These signals are recognised by Toll Like Receptors (TLR). This activates the acute phase cascade and LXR/RXR (Figure 1B) resulting in infiltration of immune cells (macrophages, neutrophils, natural killer cells) with downstream consequences involving parylation, atherosclerotic activity, apoptosis, and a potential for fibrosis (Figure 1B). Oxidative damage and breakdown products from immune cells, apoptotic cellular debris as well as pathogenic products such as LPS are solicited via nitric oxide (NO) and reactive oxygen species (ROS) in macrophages (observed in TI tissue Figure 1). Macrophage release of NO and ROS was shown to be significantly modulated across all comparisons in acute and chronic contexts with particular activation in resilient compared to acute SARS infection; and concomitant activation of receptor mediated phagocytosis in macrophages and monocytes in resilient compared to acute SARS infections (Figure 3A,B). High expression is associated with a favourable clinical endpoint in this context for mild disease (Figure 3B). Activation of the DAMP molecule, fMLP (shared by mitochondria and bacterial proteins) and fMLP receptor pathways, inducing chemotaxis, degranulation and ROS production in neutrophils was also observed in COVID cases with highest levels observed in resilient compared to persistent cases (Figure 3A), while the receptor FPR1 was significantly raised in inflamed tissue (*p* = 0.02) compared to control and NI tissue in IBD patients. The stress response involves the management of ROS and superoxides; with superoxide radical degradation pathways differentially activated in inflamed compared to non-inflamed tissues (Figure 1). Similarly, SARS infection caused a modulation of cell fate in endoplasmic reticulum stress through the proteasomal degradation system involving BAG2 signaling (Figure 1B, *p* < 0.0001; Figure 3B) and is further demonstration that both chronic and acute disease processes manipulate similar pathways to gain advantage over infection in mild cases. Significance was high across both acute and chronic conditions, but the activation z-score was heightened in acute SARS infected cells (Figure 3).

Energy is the essential currency required for cellular recovery. It has a fragile association between oxidative metabolism (mitochondrial membrane generated ATP), to fermentative metabolism (generation of lactate via glycolysis). Our data highlights a significant increase in glycolysis and glucose-6-phosphate signalling (Figure 1) with some discrepancy between proteome and transcriptome related to changes in transcripts between non-inflamed and control tissues. Glutaryl-CoA degradation, and Pentose Phosphate Pathway signalling are also significantly activated in IBD compared to control biopsied samples. Both Glutathione biosynthesis and Glutathione Redox Reactions were significantly modulated across acute infection and chronic IBD (Figure 1B, *p* < 0.0001), and glycogen degradation pathways were also affected with a significance of *p* < 0.001. These processes maintain the homeostatic balance of Nicotinamide adenine dinucleotide (NAD^+^) involved in central energy metabolism. The NADome has an immunomodulatory function [14]. NAD^+^ energy homeostasis is influenced by the degradation of the essential amino acid Tryptophan. During infection PARylation and the cellular defense regulator NFR2 (Figure 1 and Figure 3) consume the cellular pools of NAD^+^ [15]. Further demands on NAD pools are made by CD38, TLR4 signaling, and macrophage polarization which can lead to activation of the inflammasome.

As a result of these connections to tryptophan metabolism, a targeted approach was developed. Often leak precedes inflammation and thus we wanted to establish any correlations between cellular leak and tryptophan metabolism. In this study we therefore also analyzed sera from 16 patients with IBD, categorized by their confocal endoscopic leak score, endoscopic severity, and inflammatory status [16] given the significant (*p* < 0.0001 Figure 1) involvement of the extracellular matrix (ECM), integrin binding, activation of actin-based motility, and the remodeling of epithelial adherens junction in TI tissue and in NE swabs (Figure 1 and Figure 3). We observed (as in IBD) actin regulation and manipulation of junction proteins is a common feature in COVID infection, leaving cells vulnerable at local infection sites due to increased cellular permeability.

### 2.2. Tryptophan Metabolism via Kynurenine Pathway Is Altered in Immune Triggered Disease

Tryptophan metabolism plays a decisive ‘hub’ function in the management and recovery from illness. The key proteins and metabolites that establish both activity and pathological mechanisms underpinning the resolution of disease are shown in Table 1. The essential amino acid tryptophan is obtained by diet with ~95% L-tryptophan metabolised in the body and ~5% metabolised by the microbiome [17]. Its metabolism generates both energy through NAD production as well as the neuromodulators serotonin, kynurenic, quinolinic (QA), and picolinic acid (PA). Tryptophan depletion via type III pathways is the general pathway converting tryptophan to other compounds (Figure 1). Tryptophan depletion can occur through ligation of tryptophan to its cognate tRNA during translation via tryptophanyl tRNA synthetase (aptly known as WARS). Rapid secretion of WARS results from pathogen infection and serves to prime the innate immune system, bind to macrophages to induce phagocytosis and chemokine production. WARS has been demonstrated to have as much as an 18 fold increase 2 h post infection [18]. In addition, WARS has an immunomodulatory function with the capacity to be hijacked by pathogenic invaders seeking to evade intracellular detection via control of T-cell proliferation, followed by exhaustion [11,19]. Tryptophan depletion has been shown to arrest neutrophils, CD8 and CD4 T cells, and activate macrophages. Inflamed IBD TI analysis showed significantly elevated levels of WARS transcripts (Table 1). WARS was heightened in serums of active IBD and quiescent states compared to healthy controls. It was also increased in patients with high leak (CLS > 9) and with an endoscopic severity ≥1. WARS was increased in acute COVID positive cases and persisted in 3 wpi cases. It was also found to be significantly increased in COVID positive serums of older patients (>48 years of age; *p* = 0.002) and in persistent cases (Supplementary Figure S1), and patients categorised as critically ill (Table 1, and [20]). WARS was increased in inflammation during mild COVID contexts with a trend to L-tryptophan reduction also observed in inflamed IBD tissue and a significant activation of the T-cell exhaustion pathway (Repository data-IPA transcriptome). T-cell exhaustion in critical SARS COVID infection in a number of studies has been recorded [20,21], establishing that senescent T-cells and their Treg subtypes contribute to T-cell exhaustion is a feature in mild and critical cases of COVID infection, long COVID, as well as in autoimmune chronic disease such as IBD. This altered Treg capability could render protection against secondary infections or cancers impaired.

The next rate limiting step is the conversion of L-tryptophan to kynurenine (Kyn) via the anti-inflammatory enzymes IDO1, TDO (liver specific) induced by IFNγ, and the lesser understood pro-inflammatory IDO2 induced by the Aryl Hydrocarbon Receptor (AHR). We targeted the levels of IDO2 due to its association with the pro-inflammatory state. IDO2 was significantly increased (*p* = 0.024) in CD TI tissue in paired inflamed tissue; and 2 fold increase in IDO2 was observed between active disease and remission CD serums. IDO2 was not significantly altered by leak, nor endoscopic severity in our study. Paradoxically however, 4-fold reduced protein abundance of IDO2 was observed in paired biopsy samples and this was significant (*p* = 0.024 Inflamed versus non-inflamed). This trend was repeated in serums of leak patients compared to controls. Heightened amounts of IDO2 were observed in persistent COVID disease. From a metabolic standpoint the overall IDO (both IDO1 and 2) activity can be surmised by the sum of downstream metabolites anthranilic acid (AA), 3-hydroxy anthranilic acid (3OHAA), kynurenine (KYN), kynurenic acid (KYNA), PA and QA. In COVID samples, IDO activity was significantly increased for all infected samples compared to negative cases: acute infection (*p* = 0.000001), persistent disease (*p* = 0.0052); and levels remained high at all 3 wpi samples (Table 1, and Repository Data).

AA is conventionally established to be biologically inactive [23]. High levels of AA diminishes the conversion of D- to L-amino acids by D-amino acid oxidase (DAAO) [24]. Observations of increased AA in both acute SARS infection and in sera of high leak IBD patients (Table 1) signifies that AA has the potential to cross epithelial barriers into systemic circulation and inhibit the actions of DAAO, and thus has the capacity to be both a chemo-attractant to pathogenic opportunists (e.g., quinolone signal [25]) and the ability to limit DAAO H_2_O_2_ and myeloperoxidase (MPO) production in phagocytic cells, neutrophils and natural killer cells leading to long term evasion and viral dormancy within the host. Thus, AA has the potential to be used as a marker of infection severity and longevity.

The conversion of 3-OH-kynurenine to 3OHAA is done by kynureninase (KYNU). Targeted analysis of KYNU showed increased levels during active IBD disease and with increasing endoscopic severity, while it was reduced in transcripts of paired non-inflamed TI biopsies. It was not significant in differentiating leak patients. In mild cases of COVID from NE samples it was not significantly altered. However, critical COVID patients had increased levels of KYNU in serum with a marked reduction in the serum of mild COVID patients compared to control patients (Table 1 and [20]).

End products in the pathway include PA, QA, and Glutaryl-CoA. Significance (*p* < 0.01) was observed in our previous study of total PA and QA levels in IBD patients [26], while PA and QA acid levels were significantly altered between COVID naïve control cases and acute cases of COVID infection (*p* = 0.0027, 0.0013 respectively). Persistant cases also demonstrated raised levels of QA, while PA remained high in all 3 wpi (Table 1). The neuromodulatory metabolites kynurenic and quinolinic acid are prime suspect in the induction of ‘brain fog’ as a lingering consequence in SARS2 infection, and memory dysfunction or delirium, experienced during critical illness [27,28]. It is possible that locally produced neurotoxins are able to cross along the olfactory pathways and trigeminal nerves to the brain [29].

Metabolites of the KP pathway including tryptophan, and the nicotimamide (NAM) variants including Nicotinamide Riboside (NR) and Nicotinamide Mononucleotide (NMN) feed into the NAD^+^ salvage pathway (Table 1). PARP1 is a significant consumer of NAD^+^ and contributes to chronic inflammation [30]. Targeted analysis of PARP1, poly/mono levels of ADP ribose (ADPR), metabolites of CD38, as majority consumers of NAD^+^ showed significant increase in PARP1 transcripts in CD TI biopsy tissue (*p* = 0.02) with similar trends observed in protein abundances in inflamed compared to non inflamed tissue. CD serums showed increased levels of PARP1 in both active and quiescent states of the disease compared to controls. In COVID samples, Patel [20] was able to demonstrate a significant difference in the levels of PARP1 in critical disease states of infection (Table 1). Other studies have also identified PARP1 as contributing to COVID infection status [31]. Here, NE swabs of COVID infected patients showed significant increase in ADPR in acute positive cases that remained high 3 wpi, while NAM was significantly depleted from acute to almost control comparable levels in 3 wpi resilient cases. This finding has relevance as the direct conversion of NAD to ADPR by CD38 has a profound effect on induction of pro-inflammatory cytokines (IL-1β, TNFα, IFN-γ) by NK cells, activated B- and T-cells, proliferation of T-lymphocytes and provides some protection from apoptosis. Defective CD38 signaling in neutrophils has been shown to result in bacterial infection with impaired innate immunity [32].

### 2.3. Hijacking of Nuclear Transcription Factors Alters the Immune Landscape

Nuclear trafficking relies on molecular switches involving Rho-GTP binding proteins responsible for cell migration, cell-cycle progression, and phagocytosis [33]. In chronic disease, RAN (I v con, I v NI tissue) and Rho family GTPases (NI v con) were activated; while inactivation of the RhoGDI cytosolic counterpart (NI v con) can be observed (Figure 1). RAC activation was increased in acute relative to control NE samples (Table 1) and is a known inhibitor of Rho activity through the release of ROS [33].

Similarly, the nuclear receptors involved in Xenobiotic metabolism were found to be significantly activated: AHR, constitutive androstane receptor (CAR), and pregnane X receptor (PXR) are ligand activated transcriptional factors (Figure 1 and Figure 3), along with nuclear factor E2-related factor 2 (Nrf2) signaling pathway (Figure 1 and Repository IPA data) are activated in inflamed IBD tissue transcripts relative to control tissue. Targeted analysis of the AHR protein had a mean 8-fold difference in active disease relative to control tissues (Repository data). The AHR receptor is implicated in autoimmune diseases [34] but is less well understood in its role in viral infection. AHR receptor signaling was observed in SARS infected serums in critical patients [20], and tissue biopsy (*p* < 0.05). However, it could not be confidently measured from NE sampled PRM experiments in mild disease. The AHR receptor is promiscuous with a variety of ligands from both endogenous sources (kynurenine) and the microbial metabolite indole and its derivatives (bacterially synthesized from tryptophan), environmental pollutants being able to effect translocation of AHR with its ligand to the nucleus where it controls expression of the AHR repressor, and Cytochrome P450 enzymes (CYP). Of note are the modulated expression of the CYP enzymes, contributing to mitochondrial dysfunction which are observed in these samples (Repository data IPA). Ligand activation of the receptor both aids and abets inflammation depending on context. Microbial indole production in the gut activates the ligands AHR and PXR and plays a protective role in epithelial barrier function and is associated with increased IL22 and mucosal protection by increasing tight junctions and goblet cell muc2 production [34]. The enzyme DAAO catalyzes the production of indole-3-pyruvic acid from D-tryptophan, capable of activating AHR [35]. Commensals and host can compete for tryptophan locally, modulating local immunity without a systemic effect [36]. Additionally, AHR activation and translocation can be achieved in a non-canonical manner in which the AHR receptor heterodimerises with other nuclear factors such as NFkB, Estrogen Receptor (transcriptionally activated, Figure 1), Glucocorticoid receptor, the STAT protein, and C/EBP. Thus, AHR role in the immune response holds significance in both canonical and non-canonical settings.

HCAR3 was found to be increased in patients with IBD, diagnosed gut permeability and higher endoscopic severity. HCAR3 was also significant in active IBD and remission disease states compared to control (*p* = 0.025); levels of the HCAR3 ligands, 3-hydroxyoctanoic and 3-hydroxydecanoic acids were also increased (Table 1). Both PXR and CAR receptor signaling also has the function to increase vascular permeability where it was reported to be reduced in inflamed non ulcerated CD patient tissue compared to healthy control [37], and a hypersensitive immune response in patients with compromised immune barrier function resulting in increased T-cell localization and a subclinical perpetuation of leak [37]. Translocation to the nucleus mediates repression of CYP2B, gluconeogenesis, G6Pase and lipid oxidation. PXR and CAR receptor signaling facilitates ubiquitination and protein degradation by recruiting E3 ligase [37], which was shown to be modulated in our study.

### 2.4. Targeting Markers Predictive of Persistent Disease in Early Immune Triggered Contexts

**Figure 4:**
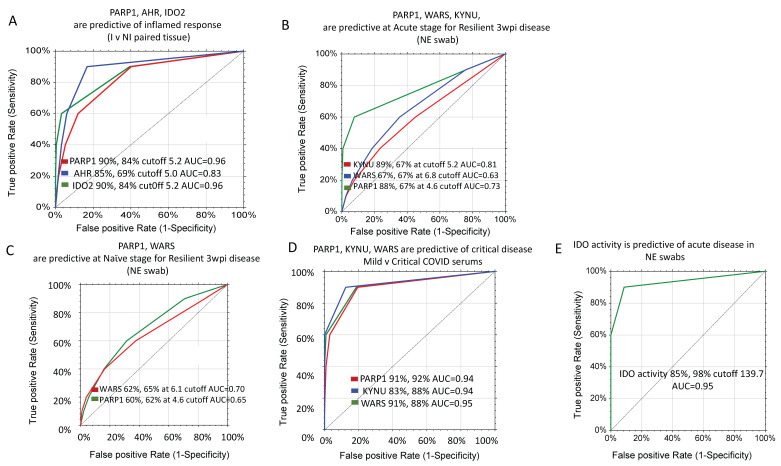
Markers of disease projection. (**A**) Inflamed paired tissue, (**B**) NE swab samples measured at Acute stage of COVID disease with known outcome at 3 wpi, (**C**) NE swab sampled at pre-COVID stage with known outcome at 3 wpi, (**D**) Critically ill COVID patients demonstrating higher abundance of predictive markers (Patel et al. [20]), (**E**) IDO activity is predictive of Acute disease and projected persistent disease at 3 wpi from both naiive and Acute NE swab samples.

The enzymes IDO2, AHR and PARP1 are predictive for the pro-inflammatory response in paired tissue biopsies (Figure 4A). For mild disease (no hospitalizations), marker levels of KYNU, WARS and PARP1, ascertained at the acute stage of infection could distinguish a more severe outcome using ROC. Binary classifiers reached 89% sensitivity, 67% specificity (KYNU); 67% sensitivity and specificity (WARS); and 88% sensitivity, 67% specificity (PARP1). Even in naϊve NE swabs, the predictive nature of both WARS and PARP1 was apparent (Figure 4C). In critically ill patients, the sensitivity and specificity were greatly predictive of disease state for these proteins (Figure 4D). In general, IDO activity as a measure of the sum of KP metabolites was predictive for more severe outcomes at both the acute and naϊve stages of COVID sampling (Figure 4E), while ADPR, cADPR, and NAM showed predictive power for infection.

## 3. Discussion

Collectively, these findings underline the complex immune dysregulation in pathogen-influenced signaling. Although severe COVID-19 is associated with marked systemic immunosuppression, local infection in epithelial tissues is characterized by hyper inflammation. Here, common hallmarks of IBD chronic inflammation and COVID inflammation are established, providing confidence in the various biologics administered successfully across both diverse manifestations of immune diseases.

Failure to eliminate the causal factors of tissue damage transforms acute conditions to chronic inflammation. ER stress and the unfolded protein response (UPR) are synonymous. It is known that the ER plays a crucial role in the pathogenesis of infectious, inflammatory, and autoimmune diseases, although the mechanism in infection is unclear. Reducing the ER stress burden by downregulating secreted proteins increases the functional capacity of the ER while EIF2α phosphorylation attenuates translation of new proteins. The UPR and host ER homeostasis modulation by pathogens can be crucial in their replication, survival and/or clearance. Virulence factors are also associated with susceptibility to IBD, epithelial cell breach, intestinal mucosa disruption and gut microbiota dysbiosis [38]. We were able to highlight the continuance of viral protein translation by stimulation of EIF4 to subvert and gain control of translation. This viral feature has also been observed for HSV-1, and EBV [39]. Viral translation in paediatric CD was also demonstrated via increased PABC1 and EIF4EBP2 [40]. IFN induced phosphorylation of EIF2α in COVID-19 positive samples [41] is consistent with our results that show EIF2 signaling activation and ER stress at 3 wpi for patients with continuing symptoms, while suppression of host translation initiation and activation of the EIF4 was observed within 24 h of positive COVID confirmation. Our data suggests that chronic ER stress contributes to the systemic universal activation of EIF2 signaling regardless of inflammatory or non-inflammatory status (especially in IBD patients) and remains activated during persistence of disease symptoms with EIF4 stimulation a potential route for subverting translational machinery for selected viral translation during acute infection.

UPR signaling pathways can also contribute to initiation of the acute phase response (APR). LPS exposure triggers a persistent APR [42]. Our proteomics results of inflamed TI tissue show upregulation of the APR signaling cascade, which aligned with increased LPS derived 3-hydroxy fatty acids in the paired leaky gut serum during (mid-high inflammation and mid-high leak). Although high acute phase protein levels have been mostly associated with bacterial infections, there is increasing evidence that APR proteins are also triggered upon viral infection [43], and that the SARS-CoV2 virus binds with great affinity to LPS to intensify inflammation even at extremely low LPS levels enhancing TLR4-NF-κB activation [44]. These results provided a novel and interesting link between excessive inflammation during SARS-CoV2 infection in patients with comorbidities who have increased levels of circulating bacterial endotoxins; and highlight the predisposition of these individuals to severe COVID-19 [44]. Our analysis shows significantly upregulated APR signaling in the COVID-19 positive swabs and that heightened levels of APR induced by the presence of local LPS stimuli from the diverse bacterial nasal population may be released into systemic circulation.

Various plasma omic studies of severe COVID-19 patients showed high level markers of gut epithelial disruption, i.e., bacterial translocation and tight junction permeability, which correlated with higher levels of systemic inflammatory markers, immune activation, lower levels of markers of intestinal function, and higher mortality rate [45]. Thus, it is not surprising that up to one third of COVID-19 patients also experience GIT symptoms, including diarrhea and vomiting, which suggests intestinal involvement during the disease course. As our results suggest, SARS-CoV2 infection, induces barrier dysfunction, and this has the potential to trigger an intestinal cytokine hyper-state with the potential for systemic involvement. Intestinal involvement may promote the disease progression through the gut-lung-axis with dire consequences [46]. Moreover, gut leakage markers and inflammasome activation have also been observed with cardiac involvement even in those with no pre-existing cardiovascular disease, suggesting a potential gut-heart axis in COVID-19 [47].

The gastrointestinal tract is an important entry and replication site for SARS-CoV2; since its main binding receptor, ACE2 is highly enriched in the intestinal mucosal glands and enterocytes [48]. Our results show upregulated actin cytoskeleton signaling and actin nucleation by ARP-WASP complex particularly in persistent COVID-19 cases and chronic non-inflamed IBD tissue, indicative of continuous tissue remodeling and immune cell activity. In the gut barrier, the actin cytoskeleton is the framework that supports its intercellular tight junction dynamics, and cell matrix adhesions in migrating cells [49]. Increased trans epithelial permeability results from pathogens liberating pore-forming toxins LPS and cytoskeleton modifying proteins. On the basal side of the epithelium, activated immune cells also induce ‘barrier disruption’ to facilitate their access to the sites of pathogen invasion by secreting reactive oxygen species (ROS), proteases, and pro-inflammatory cytokines such as TNF-α, interferons, and interleukins (IL-1) [50]. Similarly, nasal cytoskeletal integrity is crucial in curbing viral dissemination. The viral rendezvous with the cytoskeleton involves docking at intermediate filament proteins to gain entry into target cells and hijacking the microtubules for transportation to replication sites, resulting in filament polymerization [51]. Evidence demonstrates that resident microbiota influence viral load, innate immune response and clinical symptoms severity [52,53].

Neutrophils are the most abundant immune cells present at inflammation sites. The resulting ROS arrests actin dynamics. Sepsis and septic shock are also characterized by increased neutrophil protease levels, i.e., neutrophil elastase, fibrin and matrix metalloproteinases, and MPO, which results in production of WARS [54]. Aromatic D-tryptophan and D-phenylalanine may elicit a chemotactic response via activation of HCAR3 and fMLP, suggesting their synergistic action on neutrophils [55]. Our Nasal swab COVID results show activation of fMLP receptor pathways and a significant increase in levels of ADPR, NAM and cADPR to support this theory. Partida-sanchez et al. demonstrated conclusively that neutrophil chemotaxis to fMLP, absolutely depends on an extended cADPR-regulated Ca^2+^ influx; and that CD38 expression by myeloid cells is crucial for the recruitment of immune cells to infection sites and bacterial clearance [56]. HCAR3 is also highly abundant in neutrophils and macrophages, and it is activated by KYNA [57]. DAAO catalyzes the production of AHR agonists, including indole-3-pyruvic acid (I3P) though the enzymatic conversion of D-tryptophan to I3P [35], and is found in neutrophils which produce MPO and hypochlorous acid with immediate effect on the resilience to bacterial infection noted [58]. It has been suggested that AHR activation is a common corona virus strategy to evade antiviral immunity and promote viral replication [59].

Our IBD results show endogenous salvage pathways for NAD and NAD metabolites (NADome) are dominated by the tryptophan pathway as a ‘hub’ for energy homeostasis for both human, virome and microbiome support which is open to sabotage by pathogens. Activation of tryptophan metabolism, associated proteins and their metabolites; with WARS, IDO1/2, HCAR3, KYNU, kynurenine, KYNA, AA, and 3OHAA upregulated in inflamed tissue and active disease. WARS, IDO, KYNU and downstream metabolites were also upregulated in active COVID nasal swabs. Previous IBD studies have found positive correlations of IDO1, and KYNU with endoscopic sub-score, inflammation, clinical disease activity, time to surgery and hospitalization in UC [60]. Stimulation of KYNU activity in inflamed ileum CD tissue resulted in the accumulation of AA and 3OHAA and significant differences between Treg cells and neutrophil granulocyte infiltration [61]. In further studies, pathways affected during IBD progression and post treatment recovery included genes involved in tryptophan degradation, receptors of tryptophan metabolites and NAD^+^ turnover (i.e., IDO, KYNU, HCAR3, CD38 and NNMT) being synchronously co-regulated [61]. Treatment with Infliximab (TNFα inhibitor) removes excess of inhibitory NAM and helps maintain high levels of NAD^+^-dependent pro-inflammatory signaling [62,63]. While KYNU relevance to inflammation has been demonstrated in psoriasis in which epithelial cells only expressed KYNU after stimulation with IFNγ or TNFα; and combining the cytokines significantly enhanced IDO and KYNU in various cell types as much as 100 fold [64]. Unsurprisingly, anthranilate derivatives have been successfully used in topical psoriasis treatment [65]. It is conceivable that these drugs in fact inhibit KYNU.

We demonstrated that PARP1 transcript and PARP1 enzyme were regulated in inflamed tissue and in IBD leaky serum. NE swabs of acute COVID infected patients showed significant elevation of extracellular NAM, ADPR and cADPR and increase of extracellular NAD^+^, MeNAM and NMN remained high for symptomatic disease. NAD^+^ can also engage purinergic receptors triggering an inflammatory response by acting as a DAMP; and purine nucleotide biosynthesis in IBD (Figure 1A, seen in Nucleotide biosynthesis and Urea cycle) with potential for sabotage by purinolytic bacteria in the microbiome (altered in IBD microbiomes). It has been suggested that NAD^+^ release occurs by mechanisms involving active exocytosis, trans-membrane transporter diffusion in living cells or passive leakage across disrupted membranes of necrotic cells [14]. NAM is an endogenous inhibitor of PARP1 and is salvaged back to NAD^+^ while ADPR monomers are transferred onto cell proteins for DNA/tissue repair by PARPS, which absolutely depend on the NAD^+^ pool. Augmented PARP activity by ROS injury was found to be detrimental to endothelial cells; which was prevented by PARP-1 inhibitor NAM [66]. Thus, the upregulation of extracellular NAM and NAD^+^ in nasal swabs of infected subjects support a healing role. However, this should be followed up with further measurements of intracellular NADOME levels in future work.

## 4. Materials and Methods

### 4.1. Participants, Samples, and Data

Eligible participants were ≥18 years of age with written informed consent. The samples were obtained from two study groups. Study one represented cases of IBD. TI biopsy tissue from 38 patients including controls (n = 7), CD with inflammation (n = 15), CD without inflammation (n = 16) were assessed for differential expression using proteomic techniques. A further 4 paired inflamed and non-inflamed TI biopsies and 5 control age-matched samples underwent RNA Seq profiling as described [67]. Epithelial dysfunction and cellular leak from serum samples of 16 CD patients was scored by confocal laser endoscopy as previously described [16,68,69]. Study two represented COVID-19 disease. COVID naïve (control = 16), SARS-CoV2 positive confirmed (acute disease = 13), with unresolved symptoms 3 weeks post infection (wpi) (persistent disease = 6), and resolved symptoms 3 wpi (resilient = 7). All positive cases were confirmed by PCR and/or Rapid Antigen Test. The most common symptoms reported were headache, joint pain, diarrhea, loss of smell, fever and malaise. Persistent cases had ongoing symptoms and/or secondary infections. Patients in this cohort had mild disease course with no hospitalization recorded to 3 wpi. We also cross-referenced two publically available protein, metabolite, and transcription expression datasets. These data sets were attained from patients suffering mild (no hospitalization), and critical (hospitalized and requiring intensive care with mechanical ventilation) COVID symptoms. Cohort demographics are described in Patel et al., [20] for proteomic data, and Danlos et al., [22] for metabolomics data. Transcriptomic data from longitudinal time points were compared with cell-lines infected with SARS from Bojkova et al., [70]. Patient characteristics and relevant statistics are described in the Supplementary Table S1 and Figure S1.

### 4.2. Sample Preparation

Study one analysed the transcriptome, proteome and metabolome of pinch biopsies from Crohn’s disease patients undergoing colonoscopy at Concord Repatriation General Hospital. Pinch biopsies were collected from the inflamed (I) and non-inflamed (NI) portions of bowel and placed in 150 μL of RNAlater (ThermoFisher Scientific, Melbourne, Australia). Bowel was graded as either I or NI by the gastroenterologist performing the colonoscopy based on the SES-CD (Supplementary Table S2). In addition, samples were also collected from healthy control patients undergoing colonoscopy for unrelated conditions. RNA Seq analysis was used to compare expression values across 5 control and 4 paired I and NI ileal CD biopsy samples [67]. Global and Parallel Reaction Monitoring (PRM) proteomics was carried out on 100 µg protein extracted from frozen bowel tissue using a cell shearing method as previously described [71]. Serums were sampled from CD patients enrolled in the Confocal Endoscopy study measuring fluorescein leak. Protein concentration was measured and 100 µg total protein was prepared as described [16].

Study two analysed proteome and metabolome of nasal epithelium (NE) swabs. NE swabs were resuspended in 400 µL of 80% methanol with 6mg of 1.0 mm zirconium beads and used a cell shearing method [71]. Proteins were pelleted and 50 µg enzymatically treated with trypsin, while supernatant containing metabolites were stored at −80 °C until required.

### 4.3. Proteomic Mass Spectrometry of Biopsy, Serums, and Swab Samples

Mass spectrometry was carried out using a QExactive (Thermo Electron, Bremen, Germany) mass spectrometer run in DDA mode using 1.5 μg (2.0 μL from 10 μL) as previously described [71]. PRM was used to analyse all product ions resulting from specific precursor ions. PRM followed specific parent ion and transition ions of marker proteins provided in the Supplementary Table S3. The total ion current for each sample was used to normalise using relative quantitative techniques. A strict elution window of +/− 2 min and at least 3 transitions were used to remove any erroneous identifications. Peptides were eluted on an in-house manufactured 20 cm, 75 μm i.d, C18 (1.9 μm, 120 A, Dr. Maisch HPLC GmbH, Ammerbuch, Germany) column using a linear gradient of H_2_O:CH_3_CN (98:2, 0.1% formic acid) to H_2_O:CH_3_CN (20:80, 0.1% formic acid) at 250 nL min^−1^ over 60 min [67].

### 4.4. Statistical Analysis

Proteins were identified using Mascot Daemon v2.5.1 (Matrix Science, London, UK) searched against the SwissProt and SARV19 database (downloaded February 2021, containing 563,972 sequences; and July 2020, containing 271,909 sequences, respectively). Search parameters were set to carbamidomethyl (C); variable modifications, oxidation (M), phospho (STY); enzyme, semi-trypsin; and maximum missed cleavages, 1; peptide tolerance, ±5 ppm; fragment tolerance, 0.05 Da. Scaffold software (version 4.6.1, Proteome Software Inc., Portland, OR, USA) was used to compare the proteome. Peptide identifications were accepted at greater than 95% probability using the Scaffold delta-mass correction. Protein identifications were accepted with ≤1% false discovery rate (FDR) and contained at least 2 identified peptides. Expression changes across the samples were measured via spectral count, normalised to total ion count. ANOVA was used to report abundance changes controlled by the Benjamini-Hochberg procedure for multiple comparisons, *p* values set to <0.05. The studies reached a power ≥90% and was calculated using PASS software based on mean abundance values and standard deviation between groups.

The proteomic dataset of differentially abundant proteins was assessed for enriched pathways using Ingenuity Pathway Analysis (IPA^®^ Qiagen, Redwood City, CA, USA). The core analysis was carried out with only direct relationships and experimentally observed confidence considered, based on the IPA knowledge base (genes only) [72]. The *p*-value for the correlation between identified proteins and a given canonical pathway was calculated by Fisher’s exact test.

Targeted proteins were analysed using Skyline Software [73] and peptides accepted based on retention time and sequence with at least 3 transitions required. Peak area under curve of the parent ion was used to assess relative abundance of the marker panel. Log2 transformed data was evaluated using Student *t*-test, and Receiver Operating Characteristic (ROC) probability curves to measure ability to distinguish between binary classifiers.

### 4.5. Quantification of Kynurenine Pathway, NAD^+^Ome Metabolites

Mixed standards and 100 µL aliquots of NE methanolic extracts were spiked with internal standard mixture containing labelled KP metabolites; and reconstituted in 100 µL of water with 20 µL aliquots injected for analysis. MRM LC-MS/MS analysis was conducted using a TSQ Vantage mass spectrometer (Thermo, Waltham, MA, USA) connected to Vanquish (Thermo-Dionex, Thermo, Waltham, MA, USA) solvent delivery/autosampler system. Chromatographic separation was achieved using a Kinetex™ PFP column (150 mm × 2 mm, 1.7 μm, 100 Å, Phenomenex, Torrance, CA, USA) by reverse phase gradient elution at 25 °C using a ramped gradient of 0.1% formic acid to 100% methanol by 8 min as detailed in Supplementary S1–S4 (Tables S4–S8) [41].

### 4.6. Racemic Amino Acid Analysis

Methods were adapted from Ayon et al. [74]. Briefly, 40 µL of colon biopsies extracts were mixed with ^2^H_4_-alanine as internal standard. Samples were dried and derivatised with 20 µL of 10 mM Marfey’s reagent in acetone and 5 µL of 0.5 M triethylamine and incubated at 37 °C for 3 h, and quenched with 10 µL 0.5 M HCl. Samples were diluted with 120 µL of 30 % acetonitrile in 0.1% aqueous formic acid. Samples were eluted with 70% acetonitrile in in 0.1% aqueous formic acid using Phenomenex SPE Strata-X cartridges. Eluents were dried and reconstituted in 0.1% formic acid before analysis, as detailed in Supplementary S3.

### 4.7. GC Assay of Picolinic (PA) and Quinolinic (QA) Acid in NE Extracts

GC-MS analysis were carried out using Agilent Technologies GCMS system comprising 5973 inert MSD coupled to 6890 GC oven and 7683 series autosampler. PA and QA detection followed methods by Smythe et al. [75], detailed in Supplementary S4.

## 5. Conclusions

These application driven studies demonstrate the relationship between the change in tryptophan metabolism related genes, proteins and metabolites and infection associated with (out) inflammation. The immune system uses tryptophan starvation to restrict pathogen replication, and tryptophan degradation via the tryptophan/kynurenine pathway is a host mediated event activated by interferons to produce kynurenines (precursors in de novo NAD^+^ synthesis. We have shown increased AHR ligands and molecules involving DNA repair reflect immune cell activity and are major host immune regulator. Stratification of 29 patients into persistent and resilient disease types based on levels of these tryptophan associated proteins and metabolites showed a distinct signature for persistent disease, and more critical disease outcomes in a further 158 patients. Tryptophan metabolism acts as the ‘hub’, with direct metabolic products, enzymes and proteins associating with change in immune status and the potential for the marker panel to be established as a predictor of persistent disease and severe outcomes. We have demonstrated that KYNU and the requirement for tryptophan via WARS, association of the transcriptional activators AHR and HCAR3, along with ADPR and PARP1, are sufficient drivers for persistent immune disease. In TI tissue biopsy samples PARP1, DAAO, HCAR3 and WARS remained high despite remission in IBD patients. Additionally, proteins influenced by cellular permeability including KYNU, and HCAR3 induced in inflammation and active disease. These protein markers alongside relevant metabolites of the KP pathway, AA, ADPR, and cADPR signify a collection of indicators of immune disruption applicable to tissue, serum and nasal swab sampling relevant for immune triggered diseases in both COVID-19 and IBD. There are many similarities between these acute and chronic (COVID and IBD) conditions, and other autoimmune diseases. Therefore, other autoimmune conditions such as psoriasis, cystic fibrosis, sepsis as well as other viral and bacterial infections such as hepatitis, and tuberculosis may also benefit from early assessment using the marker profile.

## Figures and Tables

**Table 1:** Tryptophan pathway metabolites. Analysis type refers to transciptome (T), proteome (P), and metabolome (M). The tick symbols visually differentiate between the sample type (black circles), and for analysis type (white circle).

Name	Sample Type			Analysis Type			Observations IBD	Observations COVID
	Biopsy	Serum	Nasal	T	P	M		
**WARS ^#^**	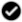	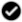	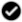	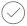	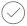		Highest in Inf v Con (*p* = 0.03) Inf v Non Inflam (*p* = 0.03) Increased in Active disease Increased in Remission Increased in Severity	Increased in Acute v naïve Increased in Persistent disease v naïve Increased in Acute Resilient v Acute Persistent (Pre-COVID) Increased in Neg Resilient v Neg Persistent Increased in Critical v Con (*p* = 0.00003 [16]) Increased in Mild v Critical (*p* = 0.0000001 [16])
**IDO2**	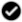	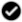	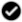	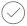	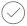		Highest in Inflamed (*p* = 0.024)	Lowest in Naïve Highest in Persistent v naïve Highest in Acute v naiive Higher in Resilient v Persistent
**Anthranilic Acid**	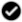	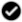	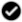			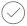	Increased in IBD Increased with severity SES (*p* = 0.05)	Highest in Acute (3OH AA Acute v naïve *p* = 0.01) Highest in Critical (*p* = 0.0017 [22])
**Kynureninase**	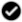	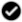	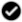	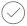	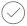		Depleted in non-inflammed IBD Increased in Active IBD Increased in severity	Highest in Acute v naïve (*p* = 0.04) Increased in Persistent disease Depleted in Acute Persistent v Acute Resilient Increased in Critical v mild (*p* = 0.0004 [16])
**Sum of all KP’s**	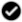	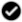	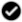			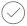	Increased in all IBD	Depleted in naïve Increased in Acute v naïve (*p* = 0.000001) Increased in Persistent v naive (*p* = 0.005) Increased in Resilient v naïve (*p* = 0.0001)
**Quinolinic Acid**		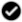	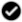			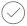	Increased in IBD	Depleted in naïve Increased in Acute v naïve (*p* = 0.0034) Increased in Persistent v naïve (*p* = 0.04)
**Picolinic Acid**		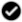	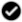			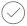	Increased in IBD	Depleted in naïve Increased in Acute v naïve (*p* = 0.0012) Increased in Persistent v naïve (*p* = 0.004)
**ADPR**			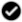			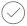	Not Done	Depleted in COVID Naïve Increased in Acute v Naïve
**cADPR**			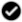			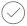	Not Done	Depleted in COVID Naïve Increased in Acute v Naïve
**NAM**			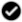			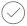	Not done	Depleted in COVID Naïve Increased in Acute v Naïve Increased in Persistent v resilient Increased in Persistent v Acute
**PARP1**	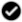		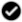	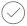	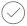		Increased in IBD transcripts I, NI v C (*p* = 0.02) Increased in IBD I v NI (*p* = 0.03) Increased in Inflamed v Con (*p* = 0.001) Increased in Remission v Con Higher in Active v Con Higher in Remission v Active	Depleted in Persistent v naïve Increased in Resilient v Persistent Increased in Acute Resilient v Acute Persistent (Pre-COVID) Increased in Neg Resilient v Neg Persistent Depleted in Mild v Critical (*p* = 0.0000001 [16])
**DAAO**	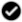	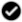		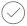	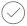		Increased in Inflamed v Con (*p* = 0.05) Increased in IBD (*p* = 0.03) Increased in Inflamed v non-inflamed Increased in Active v con Increased in Remission v Control	Not done
**AHR**	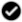	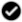		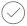	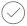		Increased in Inflamed (*p* = 0.04 transcript) Decreased free AHR Inflamed v non inflamed Increased in Active v Con Increased in Remission v con	Not done
**Tryptophan**	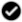	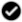	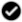			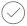	Highest in Con, increasingly depleted in NI and Inflamed. D/L Tryp % increases from Con to NI to Inflamed	Homeostatic
**3-hydroxy fatty acids**		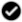				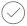	Increased in inflamed Increased in Severity High ES	Not done
**HCAR3**	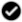	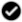	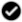		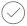		Increased in Inflamed v con (*p* = 0.03) Increased in Leak Increased in Active v Remission (*p* = 0.025) Increased in Active v Con Increased in Remission v Con	Increased in Acute v naïve Increased in Persistent v naïve Increased in Resilient v Persistent

^#^ Abundance of WARS is affected by age above 48 yrs.

## Data Availability

Detailed methods, RNA Seq, proteomic and metabolomic mass spectrometry datasets can be accessed at https://datadryad.org/stash (accessed on 14 September 2022) with doi:10.5061/dryad.rfj6q57dw and doi:10.5061/dryad.bcc2fqzgp.

## Supplementary Materials

The following supporting information can be downloaded at: https://www.mdpi.com/article/10.3390/ijms232314776/s1. Reference [75] is cited in Supplementary Materials.
