# Supplementary material for: Tryptophan Metabolism ‘Hub’ Gene Expression Associates with Increased Inflammation and Severe Disease Outcomes in COVID-19 Infection and Inflammatory Bowel Disease
Source: Int J Mol Sci. 2022 Nov 26;23(23):14776. doi: 10.3390/ijms232314776 (PMC9737535; doi:10.3390/ijms232314776)
Supplement: Supplementary file 1 [file ijms-23-14776-s001.zip › ijms-2021118-supplementary.pdf]

Table S1: Patient details for Acute and Chronic studies

	Analysis	Sample characteristics				
		**Control**	**Inflamed**	**Non-Inflamed**	**Age (%female)**	
**Chronic Study -TI Tissue IBD**	Proteomic (PRM, Shotgun)	7	15	16	19-68 (40)	**Data availability** doi:10.5061/dryad.rfj6q57dw
	Transcriptomic (paired)	5	4	4	19–68 (20)	
	Metabolomic	7	10	12	19-68 (40)	
**Chronic Study -Serum IBD**		**Control**	**High Leak CLS>9**	**Low Leak CLS<7**	**Age (%female)**	**Severity SES[a] (CLS range IBD)**
	Proteomic (PRM)	5	10	6	20-75 (50)	SES: 0=55%, 1=20%, 2-3=25% (CLS:0.58-22.49)
	Metabolomic	5	10	6	20-75 (50)	SES: 0=55%, 1=20%, 2-3=25% (CLS:0.58-22.49)
**Acute Study -nasal epithelium swabs COVID**		**Control ^**	**Acute infection*#**	**3wpi Persistent #**	**3wpi Resilient**	**Age (%female)**
	Proteomic (PRM, Shotgun)	16	13	6	7	18-87 (56%)
	Metabolomic	16	13	6	7	**Data availability** doi:10.5061/dryad.bcc2fqzgp
**Publically available datasets**		**Control**	**Mild**	**Critical**	**Reference**	
**Acute study - Blood SARS COVID**	OLINK inflammation, autoimmune, cardiovascular and neurology panels	28	26	24	Patel *et al.* 2021. https://doi.org/10.1038/s41598-021-85877-0 https://phidatalab-shiny.rosalind.kcl.ac.uk/COVID19/	
**Acute study - Blood SARS COVID**	Targeted and untargeted metabolomics.	29	23	28	Danlos *et.al.*, 2021 doi: 10.1038/s41419-021-03540-y	

* Swab collected within 24 hours PCR / RAT positive result.

[a] Simple Endoscopic Score (SES) is described in Supplement Table 1

^ Control swabs were naïve to SARS infection, asymptomatic of inflammatory indicators.

Symptoms of joint pain, fever, headache, loss of smell, gastrointestinal upset, malaise.

Table S2: Simple endoscopic score for Crohn's disease. Score is equal to the sum of all variables for the 5 bowel segments; rectum, sigmoid and left colon, transverse colon, right colon and ileum.

	Simple endoscopic score			
**Variable**	**0**	**1**	**2**	**3**
Size of ulcers	None	Apthous ulcers	Large ulcers	Very large ulcers
sDiameter of ulcers	None	0.1-0.5cm	0.5-2 cm	>2 cm
Ulcerated surface	None	<10%	10-30%	>30%
Affected surface	Unaffected segment	<50%	50-75%	>75%
Narrowings	None	Single, can be passed	Multiple, can be passed	Cannot be passed

Table S3: Proteomic MRM transition list

Compound Group	Protein name	Compound Name	Precursor Ion	Product Ion
sp|P49019|HCAR3_HUMAN	Hydroxycarboxylic acid receptor 3	STSVELTGDPNK	416.54198	358.208495
				437.219257
				473.235438
				486.753464
				504.230018
				530.256902
				617.314082
				718.361761
sp|P35869|AHR_HUMAN	Aryl hydrocarbon receptor	NDFSGEVDFR	593.262184	809.3788
				665.325316
				722.34678
sp|P23381|SYWC_HUMAN	Tryptophan--tRNA ligase, cytoplasmic	ALIEVLQPLIAEHQAR	601.015865	640.316148
				711.353262
				824.437326
				937.52139
				1034.574154
sp|Q02747|GUC2A_HUMAN	Guanylin	VTVQDGNFSFSLESVK	878.941241	896.472374
				948.442137
				1043.540788
				1095.510551
				1157.583716
				1182.542579
				1214.605179
sp|Q16719|KYNU_HUMAN	Kynureninase	VALHLDEEDKLR	479.928057	531.324922
				649.366787
				660.367515
				789.410108
				904.437051

		DVFQELEK	504.255839	619.272218
				646.340632
				732.356282
				793.409046
sp|P09874|PARP1_HUMAN	Poly [ADP-ribose] polymerase 1 (ADPRT)	WYHPGCFVK	568.770937	641.283057
				650.333044
				744.292242
				891.360655
		VVSEDFLQDVSASTK	812.906867	948.499651
				1132.552081
		LQLLEDDK	487.263664	506.209283
				619.293347
				712.387582
				732.377411
				827.414525
sp|P14920|OXDA_HUMAN	D-amino-acid oxidase	EGADVIVNCTGVWAGALQR	653.663274	900.505012
				957.526475
				1058.574154
		DTVLGFR	404.221602	429.234376
				492.292893
sp|Q6ZQW0|I23O2_HUMAN	Indoleamine 2,3-dioxygenase 2	ESGDFLYR	493.732531	713.361701
				857.415194
		TLESILHPR	533.306198	272.171716
				397.723978
				544.297704
				657.381768
				794.44068
		NLALPFVEVSR	622.853512	417.229428
				656.376623
				736.398815
				755.445037
				833.451579

				946.535643
		LPQLIDAHQLQAHVDK	913.4996	938.505406
				1016.552356
				1075.564317

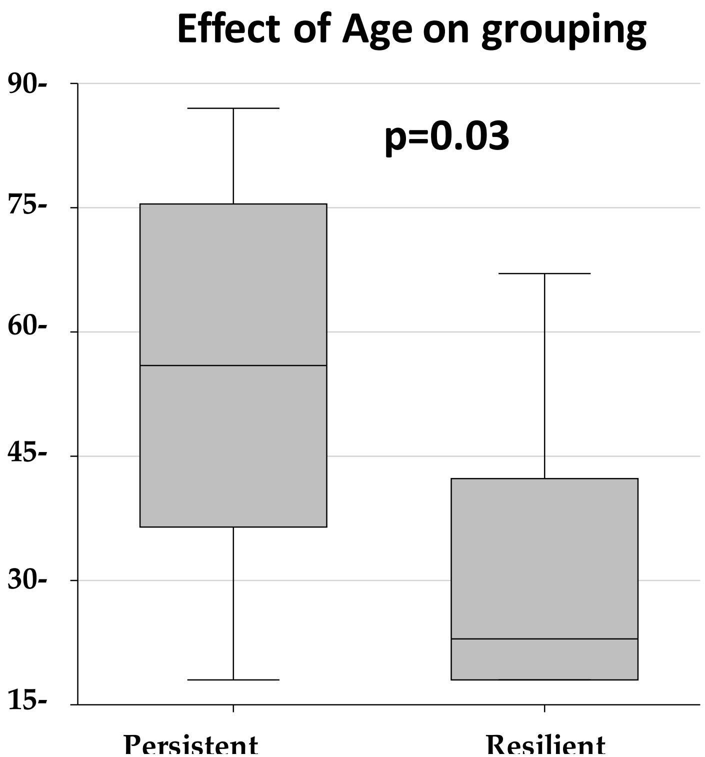

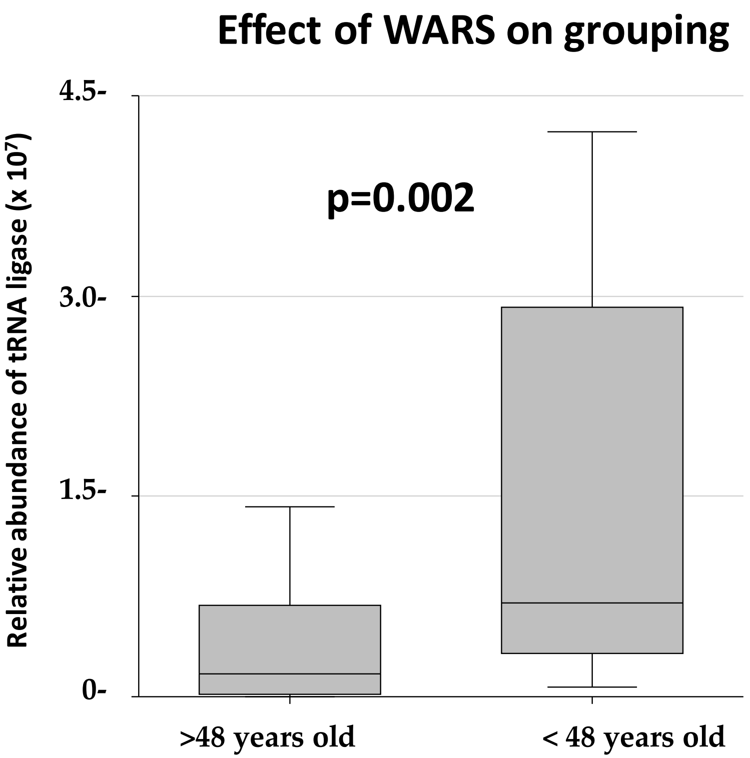

	Negative	Acute	Persistent	Resilient
Negative	1	0.18	1	0.03
Acute		1	0.43	0.35
Persistent			1	0.23
Resilient				1

Figure S1: Nasal Swab relative abundance measurements using PRM. T-tests were used to demonstrate no significant difference between age and infection, and age and persistent disease (Top panel). Groupings were separated based on cohort mean age of 48 years (above compared to below 48 years was assessed). However, resilience was a feature of age and obtained a significance level of p=0.03 (lower table). There was no significant difference between age and abundance of the markers except for tRNA ligase which was higher in samples below the age of 48 (mean age) compared to above age 48 with a significance value of p=0.002. There was no significant difference between male and female abundance of markers observed.

Acute (n=13), Persistent (n=5), Resilient (n=7), Negative control (n=21)

Supplementary S1: Detailed methods for quantitation of KP metabolites

LC-MS/MS analysis was conducted using a TSQ Vantage mass spectrometer (Thermo, USA) connected to Vanquish (Thermo-Dionex USA) solvent delivery/autosampler system. Chromatographic separation was achieved using a Kinetex™ PFP column (150mm x 2 mm, 1.7µm, 100 Å, Phenomenex USA) by reverse phase gradient elution at 25˚C. The mobile phase consisted of aqueous 0.1% formic acid (A) and methanol (B). The gradient elution was programmed as follows: start at 10 % B, hold 2 minutes, ramp to 60%B in 4min, then to 100%B in 8min. In 0.4min set to 10 % B and equilibrate for 5.6 min. Total run time is 20 min.

Mass spectrometric detection was performed using multiple reaction monitoring (MRM) with heated electrospray ionization (HESI) source in positive mode. MSD parameters were optimised using Anthranilic acid direct infusion and tune file created was used in the created method. The conditions were: ion spray voltage, 4,000 V; vaporizer temperature 300 ˚C, capillary temperature 300 ˚C, collision argon gas 1 Torr, sheath and auxiliary gas valves (nitrogen) set at 20 and 10 arbitrary units respectively. The MRM transitions for all analytes were optimised using a syringe infusion pump and are shown in table S3. Data acquisition and processing were performed with Xcalibur™ (version 2.2, 2011 Thermo Fischer Scientific, Waltham MA).

Table S4: Metabolomic transition list Kynurenine Pathway metabolites in nasal swab extracts

Metabolite	Transitions (*m*/*z*) Precursor > product ion	Internal STD for quantitation	Collision energy (V)
Anthranilic acid	138.2 > 120.2	$^{13}C_6$ Anthranilic acid	8
$^{13}C_6$ Anthranilic acid	144.4 > 126.2		8
3-OH Anthranilic acid	154.2 > 136.2	$^{2}H_3$- 3-OH-anthranilate	12
$^{2}H_3$- 3-OH-anthranilate	157 > 139		12
kynurenine	209 > 94/146.2	$^{2}H_4$ -kynurenine	20
$^{2}H_4$ -kynurenine	213 > 122/149		20
Tryptophan	205 > 118/146	$^{2}H_3$-tryptophan	20
$^{2}H_3$-tryptophan	208 > 147/190		20

Supplementary S2: NAD$^+$ome LCMS/MS assay of nasal epithelial (NE) swab extracts

Methods followed Bustamante et.al., [1]. LC-MS/MS analysis was conducted using a TSQ Vantage mass spectrometer (Thermo, USA) connected to Vanquish (Thermo-Dionex, USA) solvent delivery system/autosampler using an adaptation of a previously published method by Bustamante et al [1].

Isotopically enriched (^2H) internal standards were purchased from Toronto Research Chemicals and primary standards from Sigma-Aldrich.

HESI-MS parameters: Ion spray voltage 4,000 V; vaporizer temperature 300 ˚C, capillary temperature 300 ˚C, collision gas 1.0 Torr. These parameters were optimised using NMN solution in positive ion mode. Calibrators of known concentrations (0, 0.02, 0.04, 0.06, 0.08, 0.1, 0.2, 0.3, 0.4 µM) of NADOME metabolites were prepared by mixing aliquots of standards with a fixed volume of internal standard mixture. Similarly, NE extracts were mixed with internal std. cocktail, dried and reconstituted in 50 µl of 100 mM ammonium acetate in water. Samples were filtered into LC vials and 20µL injected for analysis. Data acquisition and processing were performed with Xcalibur™ (version 2.2, 2011 Thermo Fischer Scientific, Waltham MA). Mobile phases consisted of 5mM ammonium acetate in water pH 9.5 (A); 100 % Acetonitrile (B) according to Table S5 using a Phenomenex Luna 3 µm NH_2 100 Å 150 x 2 mm column.

Table S5: SRM transition list for NAD$^+$ome metabolites

Metabolite	Transitions (*m/z*) Precursor > product ion	Internal STD for quantitation	Collision energy (V)
NAM	123 > 78/80	$^2H_4$-NAM	20
$^2H_4$-NAM	127 > 80/84		20
Methyl NAM	137.2 > 92.2/94.2	$^2H_4$-NAM	20
NMN	335 > 123	$^2H_4$-NMN	20
$^2H_4$-NMN	339 > 127.1		20
NAD	664 > 428/524	$^2H_4$ -NAD	18
$^2H_4$ -NAD	668 > 428/524		18
NADH	666 > 514	$^2H_5$-NADH	20
$^2H_5$-NADH	671 > 514		20
ADPR	560 > 136	$^2H_5$-NADH	28
Cyclic-ADPR	542 > 232/428	$^2H_5$-NADH	28
NADP	744 > 136	$^2H_5$-NADH	30
NADPH	746 > 729	$^2H_5$-NADH	20

Table S6: Separation gradient for SRM NAD$^+$ome metabolites

Time (min)	% Mobile phase B	Flow: µL/min
0	75	300

2	55	250
2.1	40	250
7	20	250
8	5	250
15	5	250
15.1 long equilibration	75	300
25	75	300

Supplementary S3: LCMS/MS Racemic amino acids analysis of colon biopsy extracts

Methods were adapted from Ayon et al. [2], LC-MS/MS analysis was conducted using a TSQ Vantage mass spectrometer (Thermo, USA) connected to Vanquish (Thermo-Dionex USA) solvent delivery/autosampler system. Chromatographic separation was achieved using a Kinetex™ PFP column (150mm x 2 mm, 1.7µm, 100 Å, Phenomenex USA) by reverse phase gradient elution at 30˚C. The mobile phases consisted of aqueous 5mM ammonium formate pH5 (A) and acetonitrile:water (80:20 v/v) containing 0.1% formic acid (B); flow rate set at 250 µl/min.

Table S7: Chromatographic gradient for Racemic amino acid analysis

Time (min)	% Mobile phase B
0	0
2	0
10	15
13	30
14	40
16	80
18	80
19	0
25	0

Supplementary S4: SIM GC assay of Picolinic and Quinolinic acid in NE extracts

GC-MS analysis were carried out using Agilent Technologies GCMS system comprising 5973*inert* MSD coupled to 6890 GC oven and 7683 series autosampler. Chromatographic column Agilent J&W DB5-MS UI 30mx 0.25mm x 0.25µm.

Picolinic and quinolinic acid in NS extracts were assayed by GC–MS in electron-capture negative ionization mode; a very sensitive method with on-column limit of detection for QUIN and PIC < 1 femtomol on column (Smythe et al. 2003 [3]). Briefly, standards and NS extracts (100-200µl) were spiked with $^{2}H_4$ -Pic and $^{2}H_3$-Quin in 13x100mm glass cell culture tubes, and dried in a Speedvac before derivatisation with 60µL TFAA and 60µL of HFP. Capped tubes were placed in a heating block at at 60°C for 30 min to produce the hexafluoro-isopropyl esters of the respective acids. Samples were then dissolved in 80µl of toluene, washed with 1ml of 5% sodium bicarbonate and 1ml of water to remove by-products. The upper toluene layer was passed through anhydrous sodium sulphate mini columns (approx. 500 mg), into autosampler vials, 2µl were injected into the GC/MS system. Sample concentrations of Pic and Quin were calculated from the standard curves generated.
Monitored SIM ions for $^{2}H_4$ -Pic, Pic, $^{2}H_3$-Quin and Quin are *m/z* 277, *m/z* 273, *m/z* 467 and *m/z* 470 respectively.
Injector temperature 250 ˚C, transfer line temperature 280 ˚C; run time 15.2 minutes using the program below.

Table S8: GC Run conditions

Rate: ˚C /min	Temperature (˚C)	Hold time
Time 0	70	2
25	150	1
30	300	4

1. Bustamante, S., Jayasena, T., Richani, D., Gilchrist, R.B., Wu, L.E., Sinclair, D.A., Sachdev, P.S., Braidy, N., *Quantifying the cellular NAD+ metabolome using a tandem liquid chromatography mass spectrometry approach.* . Metabolomics, 2017. **14**(1): p. 15.
2. Ayon, N.J., Sharma, A.D., Gutheil, W.G., *LC-MS/MS-Based Separation and Quantification of Marfey's Reagent Derivatized Proteinogenic Amino Acid DL-Stereoisomers.* J Am Soc Mass Spectrom, 2019 **30**(3): p. 448-458.
3. Smythe, G.A., Poljak, A., Bustamante, S., Braga, O., Maxwell, A., Grant, R., Sachdev, P., *ECNI GC-MS analysis of picolinic and quinolinic acids and their amides in human plasma, CSF, and brain tissue.* Adv Exp Med Biol. , 2003. **527**: p. 705-7122.
